# Tracking the nutrition transition in Tanzania, Ghana, South Africa, and Malaysia: analysis of per capita supply of energy, protein, and fat from 1982 to 2022

**DOI:** 10.3389/fnut.2026.1790169

**Published:** 2026-07-14

**Authors:** Nyabasi Makori, Akwilina Mwanri, Daniela Weible, Ee Von Goh, Johanna Schott, Trylee Nyasha Matongera, Wendy Geza, Geoffrey Adebayo Asalu, Maxwell Mudhara, Festo Massawe, Tafadzwanashe Mabhaudhi, Joyce Kinabo

**Affiliations:** 1 Department of Human Nutrition and Consumer Sciences, Sokoine University of Agriculture, Morogoro, Tanzania; 2 Department of Nutrition Education and Training, Tanzania Food and Nutrition Center, Dar es Salaam, Tanzania; 3 Johann Heinrich von Thünen Institute - Federal Research Institute for Rural Areas, Forestry and Fisheries, Braunschweig, Germany; 4 School of Biological and Environmental Sciences, University of Nottingham Malaysia, Semenyih, Malaysia; 5 Centre for Transformative Agricultural and Food Systems, University of KwaZulu-Natal, Pietermaritzburg, South Africa; 6 Department of Industry and Service, Science and Technology Policy Research Institute, Accra, Ghana; 7 Department of Family and Community Health, University of Health and Allied Sciences, Accra, Ghana; 8 Centre on Climate Change and Planetary Health, London School of Hygiene and Tropical Medicine, London, United Kingdom

**Keywords:** dietary pattern, food balance sheet, food system, gross domestic product, nutrition transition, per capita supply

## Abstract

**Background:**

Globally, dietary patterns have shifted over recent decades due to economic growth and urbanization, contributing to nutritional improvement and emerging diet related health challenges. However, few studies have compared long-term food supply trends across countries at different stages of economic development and the nutrition transition. We examined trends in energy supply (2010–2022), protein, and fat (1982 and 2022) supply in Tanzania, Ghana, South Africa, and Malaysia.

**Methods:**

Data from the Food and Agriculture Organization - Food Balance Sheets were used to assess national-level energy supply, analyzed in Stata version 18 using descriptive statistics, correlation analysis, and linear regression to evaluate trends in nutrient supply and their association with GDP per capita.

**Results:**

Economic growth was associated with increases in food supply across the four countries. Malaysia, with the highest GDP, reported an increase in energy (2,822.8 kcal/capita/day), protein (77.4 g/capita/day), and fat (89.6 g/capita/day) supply. Ghana, despite having the second lowest GDP, reported the highest energy supply (2,863.8 kcal/capita/day) and a 101% increase in protein supply, suggesting improvement in dietary diversity. In contrast, Tanzania, which experienced the lowest GDP growth, recorded the lowest energy (2,274.8 kcal/capita/day) and protein (55.6 g/capita/day) supply. Fat emerged as the secondary energy source in Malaysia and South Africa, both exhibited high sugar supply. Across all countries, animal protein supply increased more rapidly than plant protein supply. However, South Africa experienced an 18.5% decline in plant-based protein supply, indicating a gradual shift away from traditional dietary patterns. Correlation analysis showed a statistical significant relationship (*p* < 0.05) between GDP per capita and fat and protein supply, significant association between GDP per capita and both protein and fat supply (*p* < 0.05), with correlation coefficients ranging from *r* = 0.60 to 0.94 for protein supply and from *r* = 0.51 to 0.93 for fat supply.

**Conclusion:**

Trends in per capita energy, protein, and fat supply reveal uneven changes across the four countries, reflecting different stages of economic development and the nutrition transition. The divergent trends call for country-specific strategies to promote healthier, more sustainable diets and reduce the growing burden of diet-related non-communicable diseases.

## Introduction

The global nutrition landscape is undergoing rapid changes driven by demographic shift, economic growth, urbanization, and transformation in food systems. Over the past four decades, countries worldwide have experienced significant transformations in food supply patterns, which both reflect and actively shape broader shifts in dietary habits (1). The changes are particularly evident in emerging economies, where food environments have transitioned from traditional, subsistence-based systems to market-oriented and industrialized models (2, 3). Food environments, defined as the physical, economic, policy, and socio-cultural contexts that shape food choices and nutritional outcomes, play a critical role in influencing dietary behaviors (4). Consequently, these dietary shifts have far-reaching implications for public health, food security, economic and environmental sustainability and social wellbeing (4, 5). Changes in food supply patterns can influence disease prevalence, resource distribution, and equity, underlining the need for comprehensive strategies that promote healthy, equitable, and sustainable diets.

Lower-and middle-income countries, particularly in Sub-Saharan Africa, South Asia, and parts of Southeast Asia, face a double burden of malnutrition, where undernutrition and micronutrient deficiencies coexist with rising rates of overweight, obesity, and non-communicable diseases (NCDs) (6–8). The transition of emerging economies from traditional, subsistence-based food systems to market-oriented and industrialized systems fundamentally alters food environments and supply patterns (9). This process is driven by increased income and rapid urbanization, which stimulate a greater consumer demand for processed, energy-dense foods and animal-source products. Beyond these drivers, shifts are further intensified by the increased convenience, affordability, and aggressive marketing of ultra-processed foods (10), which systematically displace nutrient dense, whole grains and plant-based diets. This shift toward diets high in fats, sugars and refined carbohydrates, elevates the risk of nutrition-related chronic diseases across all socioeconomic strata (11).

Understanding long-term trends in energy, protein, and fat supply is crucial for assessing how dietary patterns are evolving during rapid socioeconomic transitions (12). Trend analysis of food supply provides valuable insights into population-level dietary changes and offers a comparative perspective across countries. However, comprehensive longitudinal analyses comparing food supply trends across lower- and middle-income countries remain limited. This gap constrains policymakers' and researchers' from identifying the direction and magnitude of dietary shifts, evaluating the impacts of agricultural and trade policies, and anticipating emerging public health risks. It also hinders the development of targeted, context-specific interventions to promote healthier and more sustainable food environments.

Tanzania, Ghana, South Africa, and Malaysia represent a diverse set of countries at different stages of economic and nutrition transitions. This diversity provides a unique lens to examine how economic growth, influence long-term trends in energy, protein, and fat supply and shape broader shifts in dietary habits and nutrition outcomes. Tanzania (GNI per capita 2023, $1,260) and Ghana (GNI per capita 2023, $2,350) are lower-middle-income countries (LMICs) in Sub-Saharan Africa, where agriculture remains a dominant livelihood source, and challenges related to food insecurity, undernutrition, and micronutrient deficiencies persist (13). Hence, considered to be in nutrition transition. South Africa (GNI per capita 2023, $6,780) and Malaysia (GNI per capita 2023, $11,780), are classified as upper-middle-income countries (UMICs), have more advanced and urbanized food systems, characterized by high market integration, supermarket penetration, and increased supply of processed and animal-source foods (14–16); hence are in an advanced stage of the nutrition transition characterized by high prevalence of DRNCDs and signs of behavioral change (17). The four countries face rising rates of overweight, obesity, and diet related NCDs, particularly in urban populations (18). Their varying levels of socioeconomic development offer a valuable comparative perspective on how economic and social change influence food supply patterns and nutrition transitions.

While previous studies have extensively analyzed long term per capita food supply trends in high income countries (19), fewer studies have provided systematic, comparative analyses in lower and middle income countries undergoing rapid socioeconomic and dietary transitions. Examining Tanzania, Ghana, South Africa, and Malaysia allows us to capture a spectrum of nutrition transition stages from early-stage, agriculture-dominated food systems to advanced, urbanized food environments. This comparative approach enables the identification of patterns, commonalities, and differences in dietary shifts across diverse socioeconomic contexts, thereby filling an important gap in the literature. The cross-country comparisons provide insights into how economic growth and urbanization influence national food supply trends and related nutrition outcomes, extending the evidence base beyond high-income settings (19).

Therefore, this study examines long-term trends (1982–2022) in the national supply of energy, protein, and fat in Tanzania, Ghana, South Africa, and Malaysia to identify patterns, commonalities and differences in the nutrition transition across these countries. Findings will inform public health strategies and policies tailored to different development contexts. Moreover, in light of the global efforts to achieve Sustainable Development Goals (SDGs), particularly SDG 2 (zero hunger) and SDG 12 (responsible consumption and production), such evidence is crucial for designing resilient food systems that promote equitable nutritional outcomes in emerging economies.

## Methodology

### Study area

We selected four countries (Tanzania, Ghana, South Africa, and Malaysia) strategically to provide a comparative lens on the nutrition transition across diverse geographical and socioeconomic contexts. Key selection criteria included availability of consistent, long-term national food supply data spanning 1982–2022 (for protein and fat), and 2010–2022 (for energy supply), representation of different income levels and stages of economic development. Table 1 summarizes key characteristics of these countries. From a conceptual perspective, this selection enables a comparison between two lower-middle-income, agriculture-dependent economies (Tanzania and Ghana) and two upper-middle-income, more urbanized countries (South Africa and Malaysia). This contrast highlighting how differences in socioeconomic development, urbanization, and market integration shape dietary patterns and nutrition outcomes, illustrating the progressive stages of the nutrition transition. This comparative approach aligns closely with the study objectives, offering valuable insights into the determinants and consequences of dietary change under varying conditions of development and food system transformation. Each country represents a distinct stage of the nutrition transition; Tanzania and Ghana illustrate early-transition contexts, where persistent undernutrition and limited dietary diversity remain major challenges. South Africa and Malaysia reflect advanced-transition contexts, characterized by the coexistence of multiple burdens of malnutrition and rising prevalence of diet-related non-communicable diseases (NCDs).

**Table 1:** Characteristics of the four countries.

Country	Income level	Stunting (% children < 5)	Underweight (% children < 5)	Overweight/Obesity (% adults)	GDP per capita (USD, 2022)	Nutrition transition stage
Tanzania	Lower-middle	30^a^	14.0^a^	19 (women)^a^ TDHS-MIS (20)	1,192	Early transition
**Ghana**	Lower-middle	17.3^b^	11.9^b^	40 (adults, 2015)^c^	2,445	Emerging transition
**South Africa**	Upper-middle	27.0^d^	6.0^d^	68 (women)^d^	6,776	Advanced transition
**Malaysia**	Upper-middle	21.0^e^	13.0^e^	50 (adults)^e^	12,048	Advanced transition

Data in a table adopted from the following sources, ^a^Tanzania National Bureau of Statistics (NBS) and ministry of Health (19), ^b^Osborne et al. (21), ^c^Ofori-Asenso et al. (22), ^d^Unicef et al. (23), ^e^National Health and Morbidity Survey (24).

### Data source

This study utilized data from the Food and Agriculture Organization (FAO) Food Balance Sheets (FBS) to analyze long-term trends in national-level energy, protein, and fat supply (https://www.fao.org/faostat/en/#data/FBSH). The FBS are compiled by the FAO using a standardized methodology that integrates national data on food production, imports, exports, stock changes, and non-food uses such as animal feed, seed, and losses. From this information, the FAO estimates the per capita daily availability of food commodities and nutrients intended for human consumption. While FBS data do not represent actual dietary intake, they serve as a widely accepted proxy for assessing national dietary supply and identifying broad trends over time. Their consistency and international comparability make national data useful for cross-country analysis and policy-relevant assessments of food and nutrition transitions.

For the analysis of disaggregated nutrient sources, we classified protein foods into animal- and plant-based categories. Animal-based protein includes meat, fish, milk, and eggs, while plant-based protein includes cereals, legumes, nuts, and other plant-derived foods. For fats, animal fat comprises lard, butter, and other fats derived from animal sources, whereas vegetable oils include soybean, palm, sunflower, and other plant oils.

### Food balance sheets−1982 to 2022

Food balance sheets downloaded from the Food and Agriculture Organization Corporate Statistical Database (FAOSTAT) were used to assess the supply of protein and fats (in terms of g/capita/day) over the 40 years from 1982 to 2022 and the supply of energy (in terms of Kcal/capita/day) from 2010–2022. To evaluate the trend in energy, protein and fat supply over the past four decades, the years were clustered into subgroups with a range of 5 years for each group (i.e. 1982–1986, 1987–1991, 1992–1996, 1997–2001, 2002–2006, 2007–2011, 2012–2016 and 2017–2022). The total energy supply (Kcal/capita/day) for the population was estimated as the sum of energy supplied by animal and plant sources.

### Data management and preparation

Raw data on per capita energy (kcal), protein (g), and fat (g) supply were extracted for each year and country from FAOSTAT. The data were imported into Stata Version 18 for cleaning, structuring, and analysis. Country-year panels were generated using the xtset command (with country as the panel identifier and year as the time variable), which enabled the examination of time-series trends within and across countries over the 1982–2022 period. Data accuracy and reliability were assessed through several steps. First, extracted values were cross-checked against the FAOSTAT online tables to ensure consistency. Second, the data were examined for outliers and anomalies using visual inspection (line plots) and summary statistics. Implausible values were flagged and re-verified against source tables. Third, completeness and continuity were assessed across the full time series to identify any missing or inconsistent entries. All variables were standardized to common units throughout the time series: energy in kilocalories (kcal/capita/day), and protein and fat in grams (g/capita/day). This harmonization ensured comparability across countries and over time.

### Trends analysis

We analyzed national per capita supply of energy, protein, and fat using descriptive statistics, time-series plots, and regression-based techniques. For each country, mean, minimum, and maximum values were calculated by decade to summarize temporal shifts. Line graphs were used to visualize annual changes in nutrient supply from 1982 to 2022. The linear regression models were intended to estimate average annual changes over the full study period; while some patterns in nutrient supply appear non-linear, the linear specification provided a standardized measure for cross-country comparison. For the GDP nutrient analysis, we applied annual linear regressions using a panel of four countries (Tanzania, Ghana, South Africa, and Malaysia) to examine associations between per-capita GDP and nutrient supply. Lag structures, alpha levels (α = 0.05), and other statistical parameters are specified to assess trends, while recognizing that the small sample size limits causal inference and that results are intended to illustrate associations rather than establish causality. In addition, we examined GDP per capita over the period 1982–2022. Beyond assessing whether annual GDP growth rates were statistically positive using one-tailed tests, we also estimated country-specific linear regression models to evaluate the association between GDP per capita and per capita supply of energy, protein, and fat. In these models, nutrient supply was specified as the dependent variable and GDP per capita as the independent variable. Regression coefficients and 95% confidence intervals were reported to assess the magnitude and statistical significance of associations. Pearson correlation analysis was conducted to examine the relationship between GDP per capita and per-capita supply of protein and fat for each country over the study period (1982–2022), and correlation coefficients (r) were calculated to assess the strength and direction of the associations.

We estimated simple linear regression models with year as the independent variable and each nutrient supply (energy, protein, fat) as the dependent variable, to formally assess trends. The slope coefficient (β) represented the average annual change, expressed as kcal/capita/year for energy and g/capita/day for protein and fat. Both β estimates and 95% confidence intervals were reported to evaluate the magnitude and precision of trends. Serial autocorrelation was tested using the Breusch–Godfrey LM test with one lag, selected based on the rule-of-thumb floor (4(T/100)2/9) which is appropriate for time series of approximately 40 annual observations. Where autocorrelation was detected, regression models were re-estimated with Newey-West heteroskedasticity- and autocorrelation-consistent (HAC) standard errors with the same lag specification to ensure robust statistical inference. The relative contribution of aggregated food groups (e.g., cereals, roots and tubers, animal products) to total nutrient supply was assessed and visualized with bar charts to illustrate shifts in dietary sources over time.

## Results

### Energy supply

**Figure 1:**
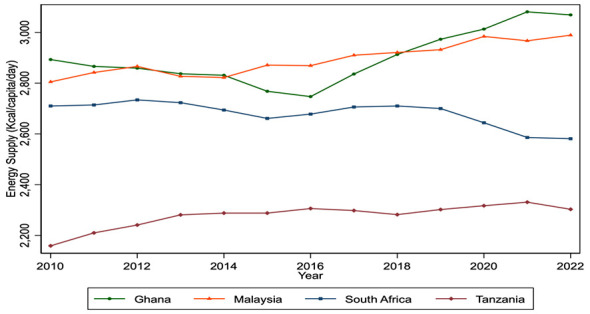
Trends of per capita energy supply across four countries from 2010–2022. Data was sourced: FAOSTAT website: https://www.fao.org/faostat/en/#data/FBSH.

Figure 1 highlights differences in both the levels and trends of per capita energy supply across the four countries. Ghana and Malaysia consistently recorded the highest energy supply levels throughout the period. Ghana exhibited a steady upward trend, particularly after 2016, peaking at over 3,000 kcal/capita/day in 2021–2022. Malaysia's energy supply remained relatively stable but gradually increased over the years, approaching 2,950 kcal/capita/day by 2022. South Africa maintained moderate energy supply of around 2,700–2,800 kcal/capita/day. The supply showed a gradual downward trend, with sharper declines after 2018, reaching 2,600 kcal/capita/day in 2022. Tanzania had the lowest energy supply throughout the period. However, the country experienced a gradual increase from around 2,200 kcal/capita/day in 2010 to 2,400 kcal/capita/day by 2021, followed by a slight decline in 2022. Ghana and Malaysia appear to be advancing more rapidly in terms of energy supply, while Tanzania remained at a lower level. Overall, the gap between the highest and lowest energy supply levels across countries was approximately 800 kcal/capita/day, highlighting substantial disparities in energy adequacy.

**Figure 2:**
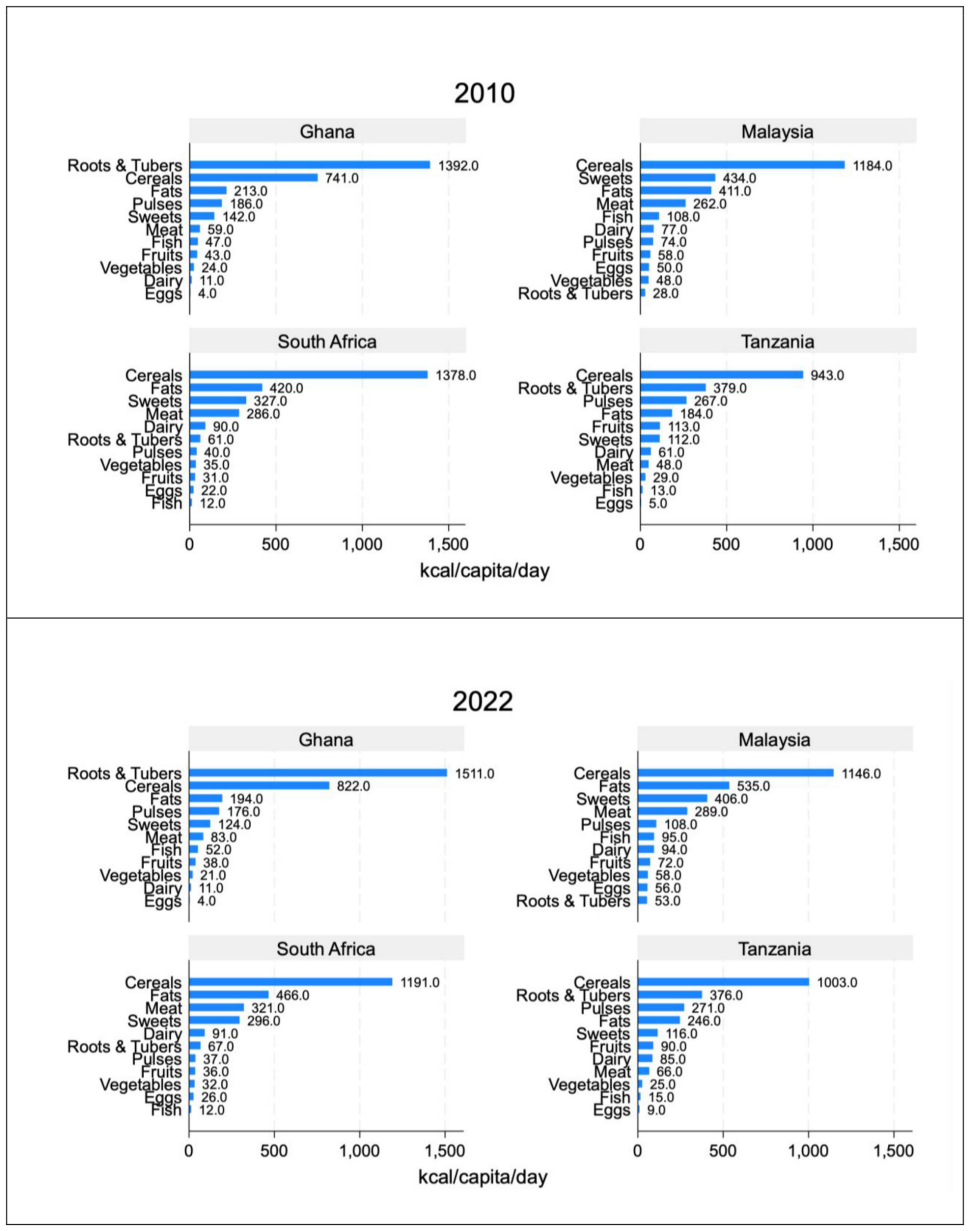
Per capita dietary energy supply (kcal) aggregated by food item in four countries for 2010 and 2022. Data was sourced: FAOSTAT website: https://www.fao.org/faostat/en/#data/FBSH.

The primary sources of dietary energy vary between countries. Ghana and Tanzania rely heavily on roots, tubers, and cereals, whereas Malaysia and South Africa have more diversified energy sources, including higher contributions from fats, sweets, and animal products. In 2022, roots and tubers provided 1,511 kcal/capita/day in Ghana (+ 4.1% since 2010), while cereals contributed 1,020 kcal/capita/day (+2.3%). In Tanzania, cereals remained the dominant source of energy, contributing 1,244 kcal/capita/day in 2022 (+5.5% since 2010), with roots and tubers supplying 812 kcal/capita/day (+1.8%). South Africa's energy supply was primarily from cereals (1,295 kcal/capita/day, −3.0% since 2010), followed by fats (448 kcal/capita/day, +6.7%) and sugars/sweets (333 kcal/capita/day, +2.4%). Malaysia recorded the highest energy contribution from sugars and sweets (437.8 kcal /capita/day), while South Africa followed closely (325.6 Kcal/capita/day). The food items that contributed the least to the energy supply were eggs in Ghana (3.9 kcal/capita/day) and Tanzania (5.5 kcal/capita/day), roots and tubers in Malaysia (42.3 kcal/capita/day) and fish in South Africa (12.5 kcal/capita/day) (Figure 2).

### Protein supply

**Figure 3:**
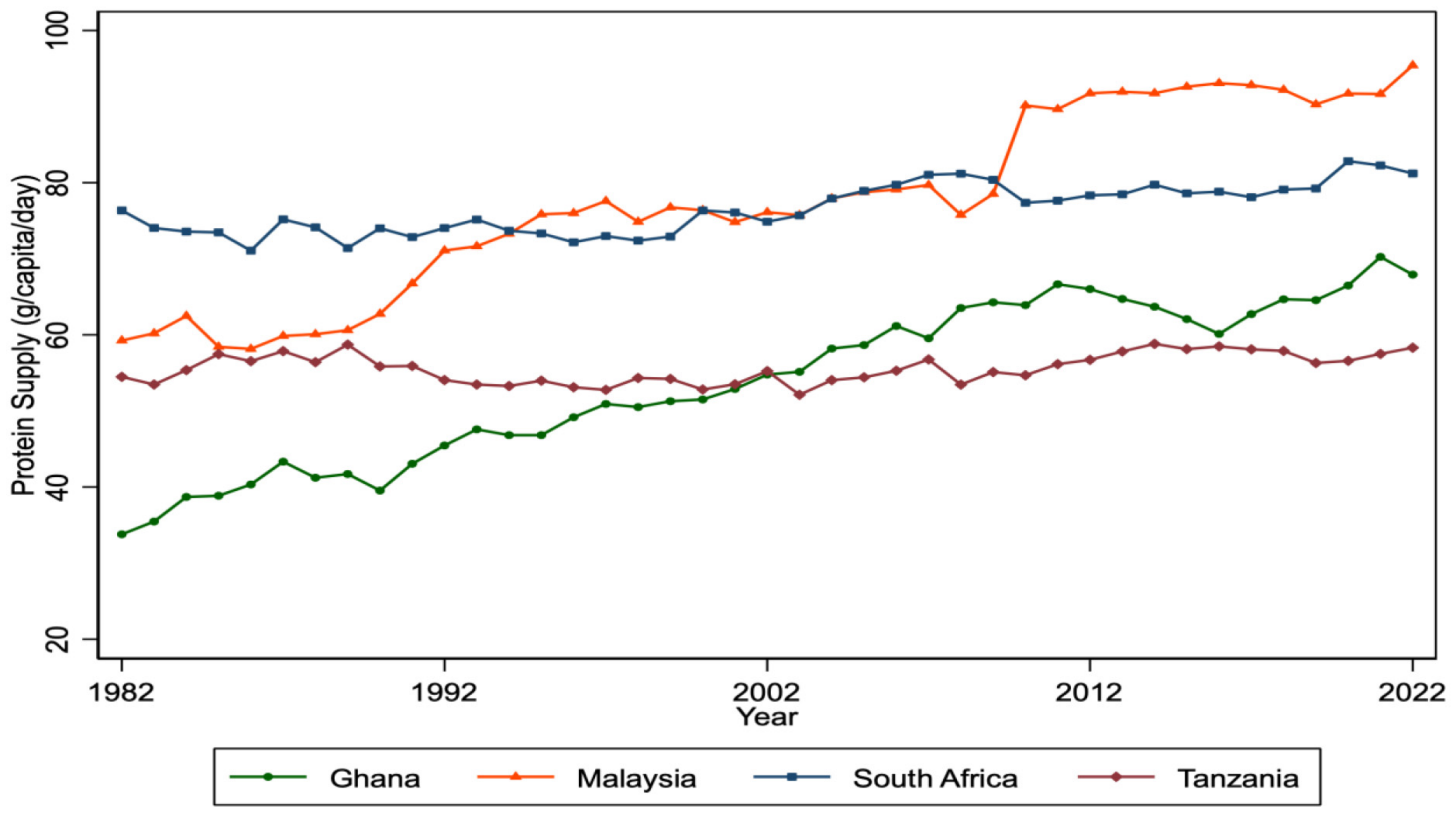
Trends of per capita protein supply across countries from 1982–2022. Data was sourced: FAOSTAT website: https://www.fao.org/faostat/en/#data/FBSH.

**Figure 4:**
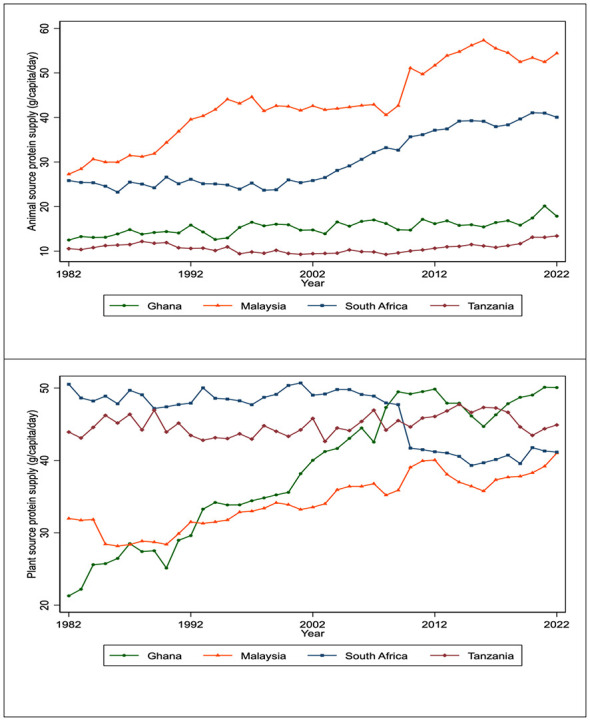
Trends of protein supply from animal and plant sources across four countries. Data was sourced: FAOSTAT website: https://www.fao.org/faostat/en/#data/FBSH.

The trends of per capita protein supply over the past 40 years in four countries are presented in Figure 3. Overall, protein supply increased by 6.3%, 6.9%, 61%, and 101 % in South Africa, Tanzania, Malaysia and Ghana, respectively between 1982 and 2022. Animal-based protein consistently grew at a faster rate than plant- based protein sources (Figure 4). The proportion of total protein derived from animal sources varied widely across countries, 98.2% in Malaysia, 73.4% in South Africa, 61.1% in Ghana and 26.5% in Tanzania. Despite this, plant protein supplies also increased by 131.5%, 28.1 and 2.2%, in Ghana, Malaysia and Tanzania, respectively while South Africa recorded an 18.5% decline. Table 2 summarizes the mean per capita supply of animal and plant protein over the 40-year, grouped into 5-year intervals. Malaysia showed the highest dependency on animal protein, the mean supply was 43.1g/capita/day, whereas South Africa, Tanzania and Ghana, relied more on plant protein (46.2 g/capita/day, 44.9g/capita/day and 38.5g/capita/day, respectively) (Figures 3, 4). Tanzania had the lowest animal protein supply (10.7 g/capita/day), followed by Ghana (15.3 g/capita/day) and South Africa (30.3 g/capita/day). Additionally, Tanzania demonstrated steady growth in animal and plant protein supply, increasing by 1 g/capita/day in each category over the 40 years' period.

**Table 2:** Per capita supply of protein from animal and plant source over the period of 40 years. SD, standard deviation.

Time period	Protein supply (g/capita/day)					
	Animal source		Plant source		Total	
	Mean	SD	Mean	SD	Mean	SD
Ghana						
1982–1986	13.2	0.5	24.3	2.3	37.4	2.7
1987–1991	14.3	0.4	27.5	1.5	41.8	1.5
1992–1996	14.2	1.4	33	1.9	47.2	1.4
1997–2001	15.8	0.7	35.7	1.5	51.4	0.9
2002–2006	15.5	1.2	42.1	1.7	57.6	2.7
2007–2011	16	1.2	47.6	3	63.6	2.6
2012–2016	16	0.5	47.3	2	63.3	2.3
2017–2022	17.4	1.5	48.7	1.4	66.1	2.7
Mean	15.3	1.6	38.5	9.3	53.9	10.5
Malaysia						
1982–1986	29.3	1.4	30.4	1.9	59.7	1.7
1987–1991	33.2	2.4	28.8	0.6	62	2.9
1992–1996	41.8	1.9	31.8	0.6	73.6	2.3
1997–2001	42.6	1.3	33.5	0.5	76.1	1.2
2002–2006	42.3	0.4	35.3	1.4	77.5	1.5
2007–2011	45.4	4.7	37.4	2	82.8	6.7
2012–2016	54.8	2.2	37.5	1.7	92.2	0.6
2017–2022	53.8	1.2	38.6	1.4	92.4	1.7
Mean	43.1	8.7	34.3	3.6	77.4	11.9
South Africa						
1982–1986	24.9	1	48.8	1	73.7	1.9
1987–1991	25.3	0.9	48.2	1.1	73.5	1.4
1992–1996	25	0.8	48.6	0.8	73.7	1.1
1997–2001	24.8	1	49.3	1.2	74.1	1.9
2002–2006	28	1.9	49.4	0.4	77.4	2.1
2007–2011	34	1.8	45.5	3.6	79.5	1.9
2012–2016	38.4	1.1	40.4	0.8	78.8	0.6
2017–2022	39.7	1.3	40.8	0.8	80.5	1.9
Mean	30.3	6.3	46.2	3.9	76.5	3.2
Tanzania						
1982–1986	10.9	0.4	44.6	1.2	55.5	1.6
1987–1991	11.6	0.5	45.3	1.3	57	1.3
1992–1996	10.4	0.6	43.2	0.4	53.6	0.4
1997–2001	9.7	0.3	43.9	0.7	53.5	0.7
2002–2006	9.7	0.4	44.5	1.2	54.2	1.3
2007–2011	9.8	0.4	45.4	1.1	55.2	1.3
2012–2016	11.1	0.3	46.9	0.6	58	0.8
2017–2022	12.2	1.1	45.2	1.4	57.5	0.8
Mean	10.7	1.1	44.9	1.4	55.6	1.9

Data was sourced: FAOSTAT website: https://www.fao.org/faostat/en/#data/FBSH.

### Fat supply

**Figure 5:**
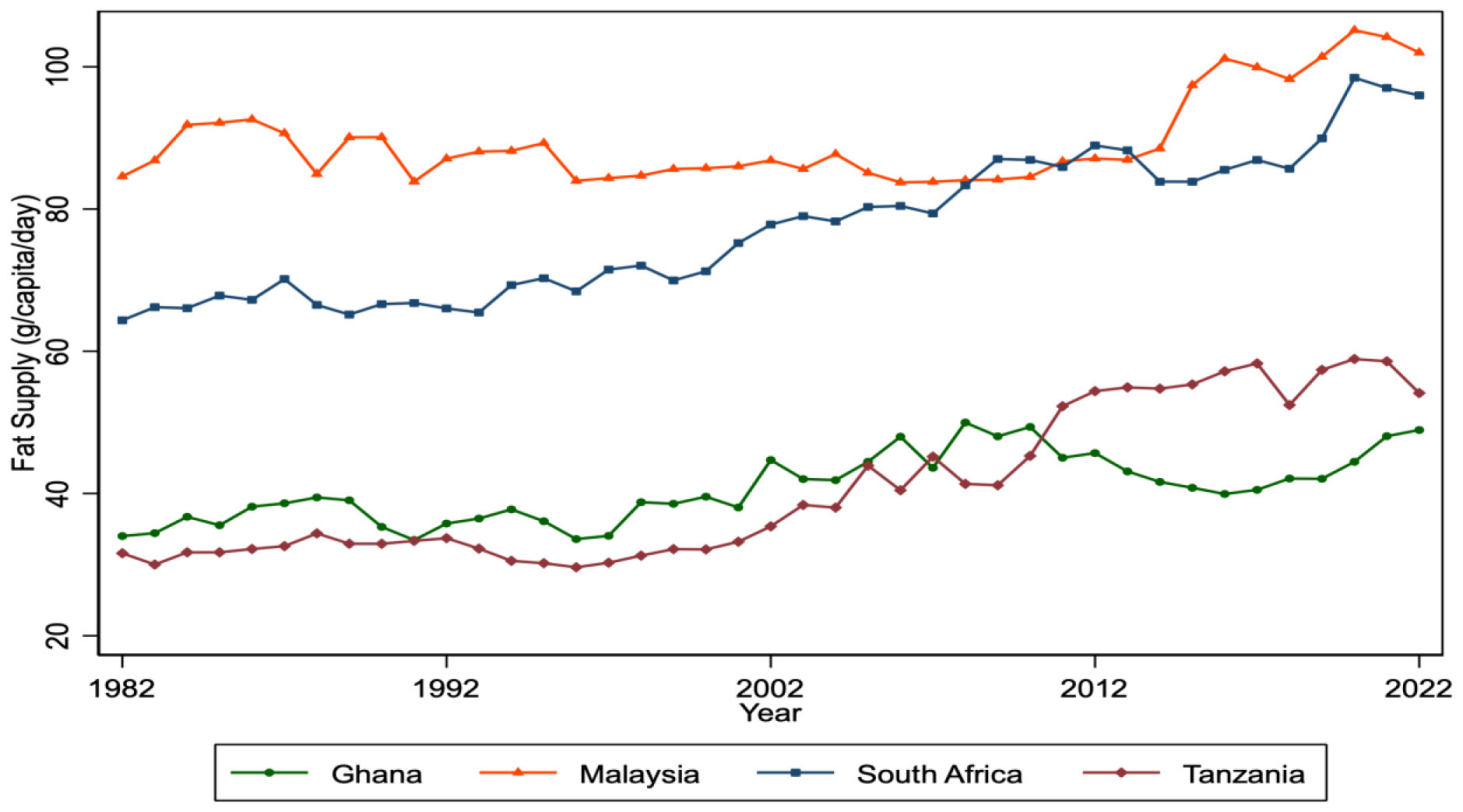
Trends of fat supply across countries 1982-2022. Data was sourced: FAOSTAT website: https://www.fao.org/faostat/en/#data/FBSH.

**Figure 6:**
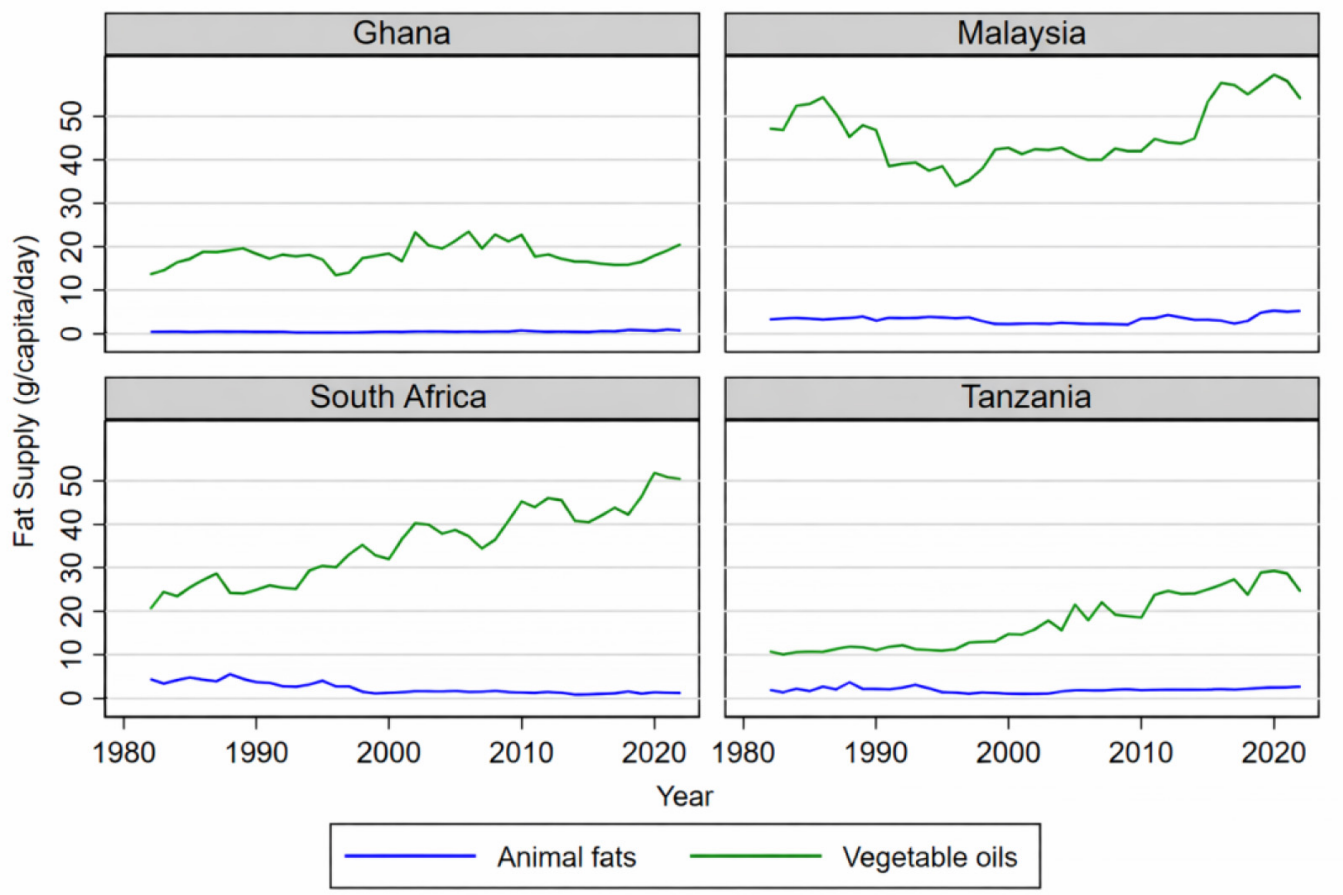
Comparison of fat supply from animal fats and vegetable oils in each country. Data was sourced: FAOSTAT website: https://www.fao.org/faostat/en/#data/FBSH.

The trends of the per capita fats supply across the four countries are shown in Figure 5. Malaysia and South Africa recorded the highest levels, exceeding 80 g/capita/day in 2022, whereas Ghana and Tanzania recorded 50 g/capita/day. Among all countries, Tanzania exhibited the fastest annual growth rates in fat supply whereas Ghana had the slowest. When disaggregated by source (Figure 6), vegetable oil supply increased steadily in all four countries, with Malaysia maintaining the highest levels throughout the period had the highest long term average supply, the mean values in four countries was 45 g/capita/day, 35.9 g/capita/day, 18.0 g/capita/day, and 17.7 g/capita/day for Malaysia, South Africa, Ghana and Tanzania, respectively. South Africa recorded the largest increase and percentage growth of 146%, followed by Tanzania (127%), Ghana (52%) and Malaysia (14%) (Table 3). For example, Malaysia's vegetable oil rose by 14% from 47.2 g/capita/day in 1982 to 60 g/capita/day in 2020. Nevertheless, South Africa has shown the highest increase in animal fat supply, at 146%, followed by Tanzania (127%), Ghana (52%), and Malaysia (14%). In contrast, animal fat supply remained relatively low and stable across countries.

**Table 3:** Annual growth in fat supply and peak supply years over 40 years in four countries (1982–2022).

Country	Increase in vegetable oil	Increases in animal fat	Highest vegetable oil supply		Highest animal fat supply	
	%	%	Year	g	Year	g
South Africa	146	71	2020	51.83	1988	5.54
Tanzania	127	42	2020	29.4	2022	2.7
Ghana	52	98	2002	23.3	2021	1.0
Malaysia	14	61	2020	59.6	2020	5.4

Data was sourced: FAOSTAT website: https://www.fao.org/faostat/en/#data/FBSH.

### Cross-country associations in fat, protein and energy per capita in four countries

Table 4 presents model results across all four countries. For fat supply, all countries showed statistically significant positive trends (*p* < 0.05). South Africa [0.79 g/capita/day (95% C.I: 0.67–0.92)] and Tanzania [0.79 g/capita/day (95 % C.I: 0.65–0.94)] showed the largest annual increase, whereas Malaysia showed the smallest annual increase of 0.27g/capita/day (95% C.I: 0.05–0.50). Ghana and Malaysia exhibited strong positive trends in protein supply, at 0.85 and 0.96 g/capita/day/year, respectively. In comparison, South Africa recorded a smaller annual increase of 0.22 g/capita/day (95% C.I: 0.17–0.28) while Tanzania showed only a marginal rise of 0.06 g/capita/day/year (*p* = 0.04). Breusch-Godfrey tests confirmed significant autocorrelation (*p* < 0.01) in all models, prompting the use of Newey-West standard errors to correct for serial correlation. Energy supply trends were positive and significant in all countries except South Africa, which showed a negative coefficient (*p* < 0.05), consistent with the decline observed from 2010 to 2022. Among the four countries, Ghana recorded the highest annual increase in energy supply (Table 4).

**Table 4:** Linear regression estimates for trend analysis per country.

Fat supply	Coefficient	95% C.I	Newey-West Std error	*p*-value
South Africa	0.79	0.67–0.92	0.061	0.001
Tanzania	0.79	0.65–0.94	0.073	0.001
Ghana	0.30	0.20–0.41	0.051	0.001
Malaysia	0.27	0.05–0.50	0.111	0.019
**Protein supply**				
South Africa	0.22	0.17–0.28	0.028	0.001
Tanzania	0.06	0.003–0.12	0.029	0.040
Ghana	0.85	0.76–0.9	0.047	0.001
Malaysia	0.96	0.85–1.06	0.052	0.001
**Energy supply**				
South Africa	−9.48	−15.04–3.92	2.525	0.003
Tanzania	10.27	5.72–14.82	2.066	0.001
Ghana	18.36	4.92–31.81	6.110	0.012
Malaysia	14.57	11.31–17.82	1.478	0.001

Data was sourced: World bank website: https://data.worldbank.org/indicator/NY.GDP.PCAP.CD

### Comparative analysis of GDP per capita growth (1982–2022) in four countries

**Figure 7:**
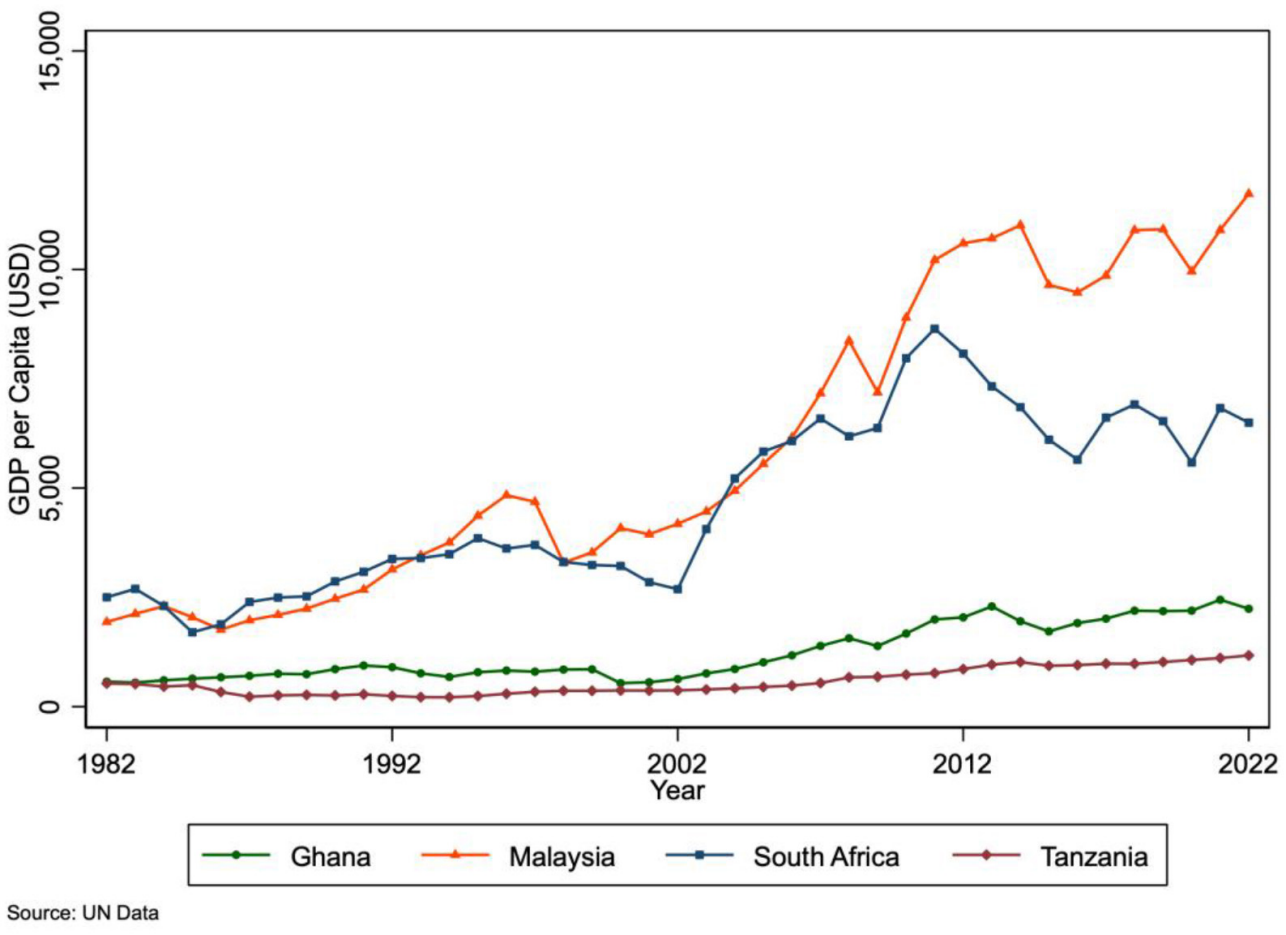
Trends of GDP growth across the countries over the period of 40 years (1982–2022).

Figure 7 presents GDP per capita trends across the four countries from 1982 to 2022, highlighting substantial economic growth disparities over 40 the year's period. Malaysia recorded the highest growth, increasing by approximately 505% from USD 1,937.7 in 1982 to USD 11,731.4 in 2022. Ghana showed notable progress, especially after 2000, reaching its peak in 2021 with cumulative growth of 292%. South Africa experienced a more modest growth of 160%, peaking at USD 8,646.0 in 2011, while Tanzania had the lowest growth rate of 121%, reaching USD1, 172.0 in 2022. Malaysia and Ghana exhibited significant GDP growth over the study period (*p* < 0.05), with Malaysia showing the largest overall increase. In contrast, South Africa and Tanzania experienced positive average growth rates, but these were not statistically significant (*p* > 0.05) (Table 5).

**Table 5:** Annual GDP growth (%) across four countries from 1982–2022.

Country	Mean growth (%)	Standard deviation	*p*-value
Ghana	4.23	12.03	0.002
Malaysia	5.18	10.63	0.016
South Africa	3.33	14.3	0.075
Tanzania	2.68	11.24	0.070

### Association between GDP per capita and supply of fat, protein, and energy across countries

**Figure 8:**
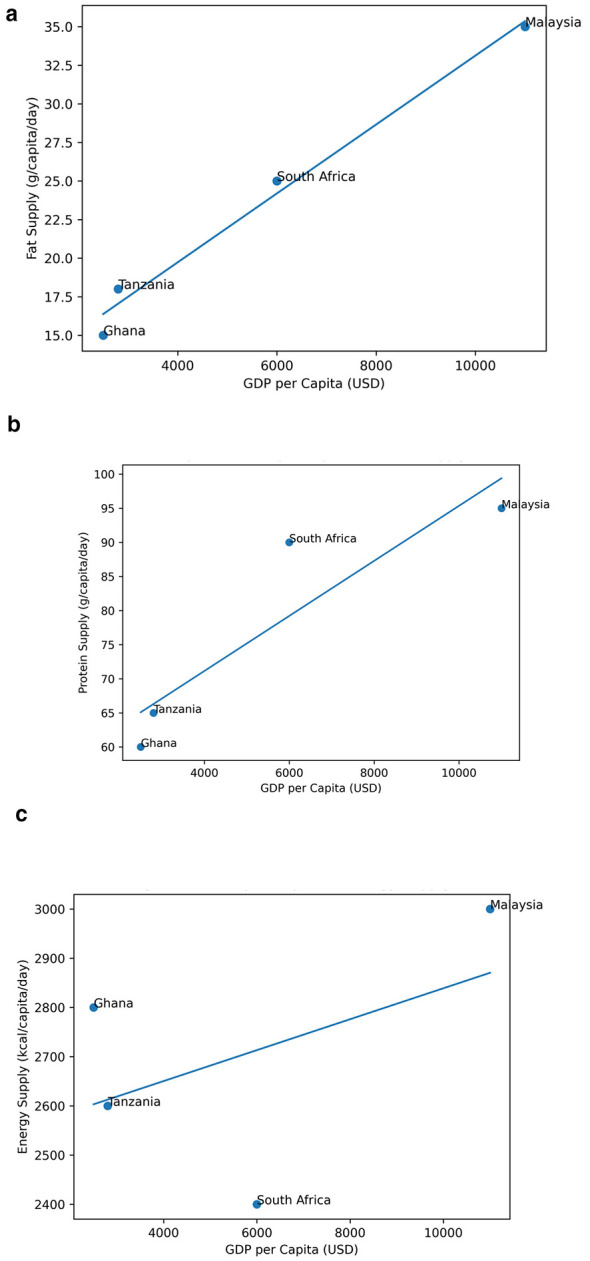
**(a)**: Association between GDP per capita and fat supply across four countries. **(b)**: Association between GDP per capita and protein supply across four countries. **(c)**: Association between GDP per capita and energy supply across four countries. Data was sourced: FAOSTAT website: https://www.fao.org/faostat/en/#data/FBSH.

Figures 8a, b, c illustrates the association between GDP per capita and annual changes in per-capita daily fat, protein, and energy supply across four countries. Fat supply increased with GDP per capita in all countries (*p* < 0.05), with the highest annual increase in per-capita fat supply observed in South Africa and Tanzania (*p* = 0.001), while Malaysia showed a lowest increase (*p* = 0.019). Protein supply increased significantly in Ghana (0.85 g/capita/day; *p* = 0.001) and Malaysia (0.96 g per capita /day/ year; *p* = 0.001), but increase was low in South Africa (0.22 g/capita/day; *p* = 0.001) and marginal in Tanzania (0.06 g per capita/day / year; *p* = 0.040). Energy supply increased significantly with GDP per capita in Tanzania, Ghana, and Malaysia (*p* < 0.05), whereas South Africa recorded a decline in per-capita daily energy supply (−9.48 kcal per capita/day/ year; *p* = 0.003).

## Discussion

### Summary of key findings

Most developing and middle-income countries are undergoing a nutrition transition, characterized by various forms of malnutrition and increasing prevalence of diseases associated with overweight and obesity. This study examined the trend of supply of energy, protein and fat as proxies to describe the trend in relation to nutrition transition. National-level food supply data used in this study may not directly reflect individual dietary intake, as actual consumption is influenced by intra-household food allocation, losses, and wastage. Findings reveal distinct yet uneven pathways across Tanzania, Ghana, South Africa, and Malaysia between 1982 and 2022. Tanzania and Ghana remained heavily reliant on cereals, roots, and tubers, with per capita energy supply reaching 2,274.8 and 2,863.8 kcal/day, respectively in 2022. In contrast, Malaysia and South Africa displayed more advanced transitions.

### Energy supply and economic growth

Countries undergoing economic and demographic changes often experience a shift in dietary energy sources from traditional staples (cereals and tubers) to more energy-dense, processed foods rich in fats and sugars (11). However, this shift does not occur uniformly across all countries and its extent and pace vary depending on factors such as income growth, urbanization, food system transformation, and cultural preferences. In some contexts, shifts are driven by increased availability and affordability of processed foods, aggressive food marketing strategies, and lifestyle changes favoring convenience over traditional diets. Trends in GDP over the past four decades provide essential context for understanding the supply patterns observed in Ghana, Malaysia, South Africa, and Tanzania. The relationship between national income and dietary energy supply is evident across these countries studied, with GDP trends shaping shifts in energy sources. Malaysia, with the highest GDP per capita, showed an increase in total energy supply, particularly from fats and sugars, reflecting a shift toward more processed and energy-dense diets (25, 26). South Africa, considered an upper-middle-income country, followed a similar pattern, indicating a dietary shift characterized by increased intake of cereals, sweets, fats, and meats. Notably, South Africa's GDP peaked around 2011, which coincided temporally with the decline observed in energy supply (Figures 1, 7). This co-occurrence suggests that macroeconomic fluctuations may have influenced national food availability and household purchasing power, highlighting the importance of economic context in interpreting nutrient supply trends (27). Economic growth and urbanization are accompanied by rapid changes in food environments and consumer behaviors, which in turn shape dietary choices, food purchasing patterns, and eating practices (28). As incomes rise and food environments become more diverse and accessible, changes in urban lifestyles encourage greater consumption of convenient, affordable, and palatable foods. Consequently, the food industry responds to changing consumer demand by increasing the supply of processed and ultra-processed foods rich in fats, sugars, and refined carbohydrates. As economies grow, food supplies tend to diversify and the availability of convenience foods increases, although the extent of this shift varies according to local food systems, policy environments, and the persistence of traditional dietary practices (29, 30).

Tanzania, has the lowest energy supply (2,274.8 Kcal per capita per day) and the lowest GDP, which reflects the characteristics of countries in the early stages of economic development, marked by persistent economic and nutritional vulnerability. Its food supply is heavily plant-based, with roots, cereals, and pulses providing the bulk of energy. Cereals, roots and tubers serve as staples for the majority of the population in rural as well as in urban areas (31, 32). As countries advance along the nutrition transition pathway, fats and sugars become increasingly prominent secondary sources of dietary energy. This reflects broader socioeconomic transformations such as improved purchasing power, greater exposure to globalized food markets, and the convenience associated with processed foods, which gradually replace traditional diets as living standards and urban lifestyles change. In South Africa and Malaysia, fat is the second most important energy source, indicating increased supply of processed and animal-based foods. The shift is further influenced by changes in food preparation practices, such as increased frying and greater use of vegetable oils, as well as the growing demand for convenience and ready-to-eat foods in urban settings. While this trend may contribute to improved overall energy availability, it also raises public health concerns due to its association with the rising prevalence of overweight, obesity and diet-related non-communicable diseases (NCDs) (16). The relatively high energy supply from sugars and sweets in Malaysia over the past decade further reflects a broader dietary shift toward processed, sugar-rich foods, illustrating how production/supply changes food environments and reshaping dietary patterns. The higher supply of sugar in Malaysia compared to global averages is consistent with broader dietary shifts toward increased supply of sugary beverages, baked goods, and processed snacks, particularly among urban and higher-income populations (14, 33). The World Health Organization recommended that the intake of sugars should not exceed 10% of total energy intake, in order to reduce the risk of diet-related chronic diseases such as obesity, type 2 diabetes, and cardiovascular diseases (34).

### Shifting protein landscapes: economic drivers and health implications

Protein is a fundamental component of a nutritionally adequate diet, playing a critical role in growth, immune function, and overall health and sometimes provision of energy (35). In the context of global dietary transitions, changes in protein supply offer valuable insights into evolving food systems and nutrition outcomes. The observed trends across the four countries over the past four decades reflect varying stages of economic development, food access, and dietary shifts. A key shift that emerged from the trend analysis shows an increasing supply of protein across all countries, though the magnitude and sources differ. The differences can be attributed to a complex interplay of factors such as economic, agricultural, cultural, and policy-related factors. Economic development plays a foundational role; higher-income countries have greater increases in animal protein supply due to improved affordability, food infrastructure, and consumer purchasing power. Empirical analysis shows that a 1% increase in GDP per capita corresponds to a statistically significant increase in animal protein consumption (36).

As household incomes rise, dietary patterns typically shift from traditional plant-based staples toward more animal-source foods; a phenomenon well documented in the nutrition transition framework (37). Malaysian high dependence on animal-based protein aligns with findings from other Southeast Asian contexts, where economic growth has enabled greater access to meat, dairy, and fish (38). Animal-source protein grew at a faster rate than plant-based protein in most cases, signaling a broader move toward more energy-dense and higher-value foods as incomes and urbanization levels rise. This trend aligns with nutrition transition theory, which explains how economic development, urbanization, and lifestyle changes drive a shift from traditional, plant-based diets toward higher supply of animal-source foods, fats, and processed products. Despite meat being a rich source of high-quality protein, excessive consumption, particularly of red and processed meats, has been associated with increased risks of chronic diseases, including cardiovascular conditions and certain cancers, raising important public health concerns (39, 40). Epidemiological evidence indicated that long-term intake of red meat exceeding approximately 100 grams per day, and processed meat consumption above 50 grams per day, is linked to a higher risk of cardiovascular diseases, certain cancers (notably colorectal cancer), and type 2 diabetes (41, 42). It is important to note that our analysis is based on national-level supply data, and not on measured individual consumption. Therefore, the health implications are interpretive and speculative, reflecting potential population-level risks rather than direct individual intake thresholds for prevention of risks. In this context, plant-based proteins are of increasing interest to consumers as alternatives to animal proteins. Proteins from plant sources offer potential health benefits, including lower cholesterol levels and reduced cardiovascular risks, and serve as a versatile and nutritionally valuable component of sustainable diets, contributing to improved public health. Moreover, from an environmental perspective, plant-based proteins generally have a lower ecological footprint than animal-based proteins thus aligning healthier eating with planetary sustainability goals (43).

Tanzania's and South Africa's marginal change in protein supply indicate persistent structural and economic barriers limiting access to diverse and animal-source foods, which may contribute to ongoing protein-energy malnutrition and micronutrient deficiencies. The variations of protein supply across countries highlight a broader inequity in protein access. Countries with greater economic resources had a higher supply of animal protein availability, while those with limited resources continue to rely on more affordable, plant-based sources (limited resources were assessed using factors such as total food supply (kcal/capita/day), and national availability of animal vs. plant proteins). Some of the key differences include total protein availability per capita, the proportion of protein from animal vs. plant sources, and accessibility of nutrient-dense foods such as dairy, eggs, and legumes. The differences highlight the need for context-specific policy responses; in higher-income or rapidly urbanizing countries, strategies might focus on promoting balanced diets with sustainable sources of animal and plant proteins; in lower-income contexts, efforts may need to prioritize accessibility and affordability of diverse, nutrient-dense protein sources.

### Raising fat supply patterns and public health implications

High supply of vegetable oil reflects Malaysia's status as a major producer and exporter of palm oil, which is widely used both domestically and globally (44). The increase from 47.2 g/capita in 1982 to nearly 60 g/capita in 2020 suggests both increased availability and possibly affordability of vegetable oils. However, the relatively small percentage increase (14%) over 40 years may indicate that Malaysia had already reached a saturation point in vegetable oil supply early in the review period. South Africa's sharp (146%) increase in fat supply may be attributed to rapid urbanization, changes in food systems, and the proliferation of ultra-processed foods rich in added fats (44–46). The fact that South Africa also experienced a decline in animal fat supply after the early 1990s suggests a substitution effect, where vegetable oils increasingly replaced traditional animal fats. This pattern indicates a dietary transition in which vegetable oils progressively substituted traditional animal fats. The shift may also be influenced by public health messaging encouraging reduced intake of saturated fats, as well as market and price dynamics favoring vegetable oils (47). The data highlight an overall transition toward greater national availability of vegetable oils, likely driven by changes in dietary habits, food industry practices, and health considerations across countries. The shift may be associated with positive health awareness on the reduction of saturated fat, especially in relation to obesity and cardiovascular risk.

In Tanzania and Ghana, the increases in fat supply (127% and 52%, respectively) were more modest in absolute terms but significant in relative terms, started from a much lower baseline, and fat supply levels remain comparatively modest. The changes likely reflect gradual transitions in dietary habits as rural populations urbanize and gain access to more commercially prepared foods. Animal fat supply, on the other hand, remained low and relatively stable in all countries, with Malaysia again leading at 5.3 g/capita in 2022. The low levels across the board could reflect a shift in consumer preferences influenced by health awareness campaigns. The decline in animal fat supply in South Africa post-1990s may also reflect economic and trade reforms that increased the availability of cheaper vegetable oils and processed foods (47). Overall, the data indicate a clear transition from animal fats to vegetable oils, driven by structural changes in food systems, urbanization, and evolving consumer behavior. Taking a closer look at the types of vegetable oil used in the four countries reveals that palm oil, which is high in saturated fat, dominates the supply. Although saturated vegetable fats are generally considered to be detrimental to health, a meta-analysis has found that this is not the case (48). The dietary shift toward unsaturated fats, particularly from vegetable oils, has been linked to positive health outcomes, including a reduced risk of cardiovascular disease. However, some randomized controlled trials revealed that the benefits of vegetable oils in lowering serum cholesterol concentrations did not translate into better clinical outcomes in terms of disease risks and survival (49). Nonetheless, policies promoting healthier oil options remain important because they can help reduce intake of harmful saturated and trans fats, improve overall dietary quality, and support population-level risk reduction, especially when combined with nutrition education and broader public health strategies (49, 50). Future policy efforts should aim to promote healthier oil options (e.g., unsaturated fats, minimal processing oil) and integrate nutrition education to guide consumer choices. Additionally, monitoring fat supply trends should be part of broader efforts to assess dietary quality and prevent diet-related chronic diseases.

### Uneven pathways: cross-country differences, and nutritional inequalities

The cross-country comparisons reveal substantial disparities in food supply patterns that highlight broader nutritional inequalities linked to economic status, agricultural capacity, and food system development (33, 51, 52). Countries with higher income levels, such as Malaysia, have experienced a marked transition in their food supply, characterized by greater availability of energy-dense, animal-source proteins, and fats, particularly from vegetable oils. This reflects a more advanced stage of the nutrition transition, where diets are increasingly varied but also more calorically concentrated and nutrient-imbalanced.

In contrast, lower-middle-income countries like Tanzania exhibit food supply patterns that are still heavily dependent on traditional plant-based staples, notably cereals, roots, and tubers. The limited diversity in food supply contributes to persistent risks of undernutrition, micronutrient and protein deficiencies, particularly in rural populations and among vulnerable demographic groups. The supply of animal-source foods in lower- and middle-income settings remains low, reflecting both limited production capacity and affordability barriers, which contribute to unequal access to high-quality protein and micronutrients.

Ghana, although a lower-middle-income country, displayed a more complex picture. Its relatively high total energy supply compared to its income level suggests some improvements in food availability, possibly due to economic growth. However, the continued dominance of cereals and root crops in the food supply still signals incomplete dietary diversification, which may limit overall nutritional quality. The increasing supply of animal protein observed in Ghana is a positive trend, yet the overall balance of macronutrients and micronutrients remains skewed in comparison to higher-income countries. South Africa sits at an intermediate point along the nutrition transition, with a food supply that is increasingly energy-dense and diverse. While access to a wider variety of food, including animal proteins and fats, has improved, there is also a notable increase in the supply of sweets. This contributes to excess energy supply, which, in combination with urbanization and sedentary lifestyles, can lead to rising rates of overweight and obesity.

The food supply trends across the four countries reflect not only economic divergence but also nutritional stratification, where supply to diverse and nutritionally adequate diets remains uneven. The inequalities are evident both between countries at different stages of economic development and within countries, where disparities in food supply and access continue to shape health and nutrition outcomes across populations. While energy sufficiency has improved in most cases, it has not translated equally into nutritional adequacy, revealing a persistent gap in the quality of national food supplies that mirrors broader structural and socio-economic divides.

### Relevance for food environments and health policy and practice

The observed differences across countries demonstrate how economic development, agricultural capacity, and cultural food preferences shape national food supply patterns. Additional factors such as infrastructure constraints, limited market access, political and governance stability, and education levels also play a role in shaping dietary patterns, though they are beyond the primary scope of this study. In Tanzania and Ghana, continued reliance on starchy staples reflects both dietary habits and the structure of domestic food production, but nutritional outcomes also depend on factors such as the availability and affordability of energy, protein, and fat, the role of crops in export markets, and their contribution to national GDP. In Tanzania and Ghana, continued reliance on starchy staples reflects both dietary habits and the structure of domestic food production, but nutritional outcomes also depend on factors such as the availability and affordability of energy, protein, and fat, the role of crops in export markets, and their contribution to national GDP. The pattern also reflects national agricultural priorities, where production systems remain heavily oriented toward staple cereal crops, with relatively limited investment in nutrient-dense foods, and animal-source products. Such production focus reinforces the dominance of carbohydrates in the national diet and constrains the availability and accessibility of diverse foods necessary for improved nutrition outcomes.

The dynamics highlight the dual burden of malnutrition that increasingly characterizes many low- and middle-income countries. South Africa and Malaysia illustrate the other side of the nutrition transition, where greater energy supply particularly from fats and oil, and sugars elevates the risk of overweight, obesity, and (NCDs). Policy responses must therefore be tailored to context and address both food production and supply. In lower-income settings, priorities should include expanding access to nutrient-dense foods such as animal-source products, legumes, fruits, and vegetables, while ensuring affordability for vulnerable populations. In middle- and upper-middle-income settings, the emphasis should shift toward moderating the supply of energy-dense, processed foods and sugars, supported by interventions such as food environment regulation, fiscal measures, front-of-pack labeling, and nutrition education. For foods prepared at home, strategies should focus on promoting healthy cooking practices, dietary guidance, and education to encourage balanced and nutrient-rich meals. At a broader level, the findings reinforce the urgency of aligning food systems with the Sustainable Development Goals, particularly SDG 2 (zero hunger) and SDG 12 (responsible supply and production), to ensure that increases in food supply translate into healthier, and sustainable diets.

### Limitations

The shorter period for energy supply limits our ability to evaluate long-term trends in energy supply relative to protein and fat over the full four decades. Consequently, comparisons of energy trends with protein and fat should be interpreted with caution, as the temporal coverage differs. Despite this, the available energy data still provide meaningful insight into recent patterns and allow for comparative analyses with protein and fat in the most recent decade.

While the study provides meaningful insights into macronutrient supply patterns across the four countries, it is important to acknowledge the limitations of the underlying dataset. The methodological changes between the older (1982–2010) and new (2010–2022), may affect both the comparability of countries and the interpretation of long-term trends.

FBS data provide average national per capita supply but do not capture within-country variability. Differences by socioeconomic group, urban vs. rural areas, or vulnerable populations cannot be directly inferred, making conclusions about dietary inequities or access speculative.

The study assessed supply data, which may differ from actual consumption due to household distribution, food loss, or wastage. Observed trends in supply do not necessarily translate to individual dietary intake or nutritional outcomes.

This study included only four countries, which limits the extent to which the findings can be generalized to other regions or global contexts. The selected countries represent specific socio-economic and developmental profiles, and therefore the results should be interpreted as context-specific comparative case analyses rather than globally representative estimates. Future studies including a broader range of countries would strengthen external validity and enhance generalizability.

## Conclusion and recommendations

The four studied countries, Ghana, Malaysia, South Africa, and Tanzania, are all at varying stages of the nutrition transition, experienced an overall increase in calorie, protein, and fat supply. However, the magnitude of supply varies according to the income levels and stages of food system transformation. The variations in stages of nutrition transition reflected combined effect on economic growth, urbanization and food system transformation, which collectively shape the pace and nature of dietary shifts, present unique challenges and opportunities. In lower-middle-income countries like Tanzania and Ghana, traditional staples still dominate national food supply and undernutrition remains a persistent challenge. However, the emerging increase in fat and energy supply also signals a growing risk of overweight and obesity, particularly among adults in urban areas.

South Africa and Malaysia exhibit a more advanced stage of the nutrition transition, characterized by dietary shift toward energy-dense, high-fat, and protein-rich foods, often associated with rising rates of obesity and non-communicable diseases, and there are also signs of behavioral change where a segment of the population is actively seeking healthier diets and lifestyles. The evolving patterns of dietary energy, protein, and fat supply across Tanzania, Ghana, South Africa, and Malaysia from 1982 to 2022 highlight the urgent need for regionally differentiated and context-specific nutrition and health policies. To effectively respond to challenges, governments must strengthen food supply policies based on country-specific needs.

In Tanzania and Ghana, where undernutrition persist, policy efforts should prioritize improving the availability, accessibility, and variety of nutrient-rich foods within the national food supply. This includes promoting the production and supply of legumes, fruits, vegetables, and animal-source foods, supported by investments in agricultural diversification and market access.

Across all contexts, including low- and middle-income countries, governments should implement regulatory measures to guide healthier food production and supply. However, the nature and emphasis of the measures may vary depending on each country's development stage, income level, and food system dynamics. For example, while South Africa and Malaysia may focus on reformulating processed foods and regulating marketing, Tanzania and Ghana might prioritize improving access to diverse and affordable nutritious foods.

In South Africa and Malaysia, the government should implement regulatory measures to guide healthier food production and supply. This may include taxes on sugary drinks, mandatory front-of-pack labeling, and limits on marketing unhealthy foods. Incentives should also be provided to encourage the supply of healthier alternatives.

Coordinated multi-stakeholder efforts are essential, with the government ensuring strong policy frameworks and adequate investment, including integrating national nutrition and food system policies that align agriculture, trade, and health objectives. Civil society and non-governmental organizations also play a crucial role in community engagement and service delivery. Media organizations can support efforts by disseminating accurate, accessible information on healthy diets and nutrition, while academic and research institutions provide the evidence base to inform, monitor, and evaluate interventions. NGOs and civil society affect consumption behaviors through outreach and advocacy, media shape consumer awareness, and research institutions generate data that inform adaptive responses to nutrition transition and changing food environments.

### Future research direction

Building on the current analysis, several actionable research directions can further strengthen the evidence base. First, since FAOSTAT reflects supply rather than intake, future studies could triangulate supply data with household budget surveys, to better understand how supply patterns translate into actual dietary behavior. This would help assess distributional effects across socioeconomic groups. Second, researchers could link long-term macronutrient supply patterns with trends in obesity, hypertension, diabetes, and other NCD outcomes using high-quality datasets such as the global burden of disease or food systems dashboard. Cross-lagged or time-series approaches could explore causality. Finally, Future research could evaluate how climate shocks, economic crises, pandemics, or geopolitical disruptions impact food supply patterns. Incorporating resilience metrics could provide insights into stability and risk management.

## Data Availability

The original contributions presented in the study are included in the article/supplementary material, further inquiries can be directed to the corresponding author.
